# Effects of whole-body vibration exercise on physical function in patients with chronic kidney disease: a systematic review and meta-analysis

**DOI:** 10.1186/s12882-023-03436-3

**Published:** 2024-01-03

**Authors:** Yan Bai, Liuyan Huang, Xiaojing Yin, Qiuzi Sun, Fan Zhang

**Affiliations:** 1 Department of Nephrology A, Longhua Hospital Shanghai University of Traditional Chinese Medicine, No.725, Wanping South Road, Xuhui District, Shanghai, China; 2 Department of Nursing, Longhua Hospital Shanghai University of Traditional Chinese Medicine, Shanghai, China

**Keywords:** Whole-body vibration, Chronic kidney disease, Physical function, Systematic review, Meta-analysis

## Abstract

**Background:**

The current state of knowledge regarding the efficacy of whole-body vibration (WBV) training for individuals with chronic kidney disease (CKD) is limited. To address this gap, the present study seeks to undertake a comprehensive systematic review and meta-analysis of clinical trials to evaluate the impact of WBV on physical function and quality of life outcomes in CKD patients.

**Methods:**

A systematic search was performed on the PubMed, Embase, Web of Science, and Scopus databases from inception to March 2023 and updated in June 2023. The inclusion criteria comprised randomized controlled studies, quasi-experimental studies, and single-arm trials that evaluated the impact of WBV on physical function, encompassing cardiopulmonary fitness, muscle strength, mobility, and balance, in CKD patients. Adverse events that were included in the study reports were recorded. The pooled evidence was assessed using the Grading of Recommendations, Assessment, Development, and Evaluation (GRADE) method.

**Results:**

Nine studies were identified, of which seven were included in the meta-analysis. The results of the meta-analysis indicated a statistically significant improvement in upper (mean difference: 3.45 kg; 95% confidence interval 1.61 to 5.29) and lower (standardized mean difference: 0.34, 95% confidence interval 0.08 to 0.59) extremity muscle strength in patients with CKD who underwent WBV training compared to baseline (low-level evidence). Furthermore, WBV training favored improved cardiorespiratory fitness, mobility, and balance function, but no statistical difference was observed. The impact of WBV training on quality of life in patients with CKD requires further validation. Notably, only one adverse event (nausea) was reported in the included studies.

**Conclusions:**

WBV has demonstrated efficacy and feasibility in enhancing muscle strength among patients with CKD. However, further investigation is warranted to determine its potential for improving cardiorespiratory adaptations, mobility, balance function, and quality of life. Additionally, future research should prioritize comprehensive reporting of WBV protocols to establish an optimal training regimen for the CKD population.

**Supplementary Information:**

The online version contains supplementary material available at 10.1186/s12882-023-03436-3.

## Key learning points

**What was known:**

1) The gradual decline of physical function in patients with chronic kidney disease because of deteriorating renal function significantly impacts their quality of life and prognosis.

2) Recently, Whole-body vibration has gained significant attention as a rehabilitative intervention for neuromuscular training.

3) To date, no quantitative analysis has been published that evaluates the impact of whole-body vibration training on physical function and quality of life among individuals diagnosed with chronic kidney disease.

**This study adds:**

1) The present systematic review and meta-analysis, comprising nine clinical trials, has demonstrated that whole-body vibration training is a significantly effective intervention in enhancing muscle strength among individuals with chronic kidney disease.

2) Despite the absence of statistical significance, whole-body vibration training appears to improve cardiorespiratory adaptation, mobility, and balance of individuals with chronic kidney disease.

3) Further investigation is required to examine the effects of whole-body vibration training on the quality of life of individuals with chronic kidney disease.

**Potential impact:**

1) The outcomes of this systematic review and meta-analysis have the potential to enhance the comprehension of healthcare providers regarding the favorable impacts of whole-body vibration training on the physical function of individuals diagnosed with chronic kidney disease.

2) The data indicate that incorporating whole-body vibration training programs into standard therapy may serve as a beneficial intervention for the physical rehabilitation of chronic kidney disease patients.

**Supplementary Information:**

The online version contains supplementary material available at 10.1186/s12882-023-03436-3.

## Introduction

Chronic kidney disease (CKD) is a global public health problem characterized by high incidence, low awareness, poor prognosis, and high medical costs [1]. In recent years, CKD has become the ninth leading cause of death in high-income countries, and its incidence is increasing yearly, with a trend toward younger people as they live longer [2]. The global loss of life expectancy due to CKD is expected to double by 2040, representing a formidable challenge for healthcare and health systems [3].

The maintenance of an individual's activities of daily living is contingent upon physical function, which is a crucial component of health-related quality-of-life assessment [4]. Patients with CKD frequently experience muscle atrophy [5], exacerbated by a sedentary lifestyle and linked to heightened morbidity and mortality rates [6]. The literature has extensively documented the correlation between muscle atrophy, reduced physical function, and decreased physical activity in CKD patients, leading to a self-perpetuating cycle [7]. Therefore, improving impaired physical function and averting physical deterioration are vital goals for maintaining the health and well-being of most of the CKD population [4].

Several studies indicate that low-cost interventions, including exercise training, may enhance physical function among patients with CKD [8–10]. While aerobic exercise, resistance training, and multi-component exercise have been proven effective, they pose a risk of injury to patients [11]. The intensity of traditional exercise rehabilitation programs may be challenging for a frail CKD population, given the potential limitations posed by the patient's clinical status or co-existing medical conditions [12].

Whole-body vibration (WBV) is a training method that uses mechanical vibration and external resistance loading to stimulate the body, causing muscle vibration and increasing central nervous system adaptations [13]. Typically, patients assume a seated or standing position on a vibration platform, which transmits exogenous stimuli of varying amplitudes and frequencies from the feet to the entire body, establishing a "skeletal-muscular" chain of connections [14]. It would be interesting to treat exercise on vibration machines as a complement to aerobic exercise or resistance training.

In recent years, WBV has become a focal point for the rehabilitation of chronic diseases [14]. Compared with other exercise modalities, WBV is not affected by subjects' motor ability and health status. Previous reports have shown that WBV induces reflexive muscle contractions in subjects, which improves perceptual-motor deficits in balance, strength, joint position sense, and muscle activity in patients with chronic ankle instability [15]; Chen et al. showed that low-frequency and high-frequency WBV had a significant effect on knee osteoarthritis patients' pain, knee extensor strength, and physical function with additional positive results [16].

To our knowledge, only Coelho-Oliveira AC et al. [17] have published a systematic review of WBV training in patients with CKD but lacks a quantitative analysis. Therefore, this study intends to collect published clinical trials and aims to answer the following questions through meta-analysis:

- 1) Can WBV training improve physical function, including cardiopulmonary fitness, muscle strength, mobility, and balance in CKD patients?
- 2) What is the impact of WBV training on the quality of life of CKD patients?

## Materials and methods

### Protocol and registration

This study was conducted and reported following the Preferred Reporting Items for Systematic Review and Meta-Analysis (PRISMA) checklist guidelines (Table S1) [18]. The study protocol has been registered on the Prospective International Register of Systematic Reviews (PROSPERO: CRD42023411120). The current study methodology is similar to the previously described protocol with a few modifications (Table S2).

### Search strategy

A comprehensive literature search was performed in March 2023 across PubMed, Embase, Web of Science, and the Cochrane Library to identify relevant literature on WBV training in patients with CKD. The search was subsequently updated in June 2023 to include newly published articles. The search strategy utilized a combination of MeSH and free-text terms, with "chronic kidney disease" and "whole body vibration" as the primary keywords. The detailed search strategy is presented in Table S3. Furthermore, relevant reviews were manually scrutinized to identify other potentially eligible studies for inclusion or citation.

### Eligibility criteria

Published studies with the following criteria were considered eligible:

- 1) Participants: adult CKD patients. Age and disease stage are not limited (pre-dialysis, peritoneal dialysis, hemodialysis, and kidney transplant recipients were eligible).
- 2) Intervention: participants in the intervention group received WBV training, whereas the control group received sham vibration or usual care. Studies comparing WBV + B versus B were also included.
- 3) Outcomes: include at least one of the following outcome evaluations: cardiopulmonary fitness, muscle strength, balance, mobility, and quality of life.
- 4) Study design: randomized controlled trial (RCT), quasi-experimental, and single-arm trial.

Studies were excluded if they met at least one criterion: 1) conference abstract, case reports, protocol, letters, commentaries, reviews, and editorials; 2) written in non-English.

### Study selection and data extraction

The search results were extracted into Endnote 20, and duplicate articles were removed. Two reviewers (YB and FZ) made the selection independently. Initially, the titles and abstracts were screened for relevance based on predetermined eligibility criteria, followed by a review of full-text articles with documented reasons for exclusion. Any conflicts were discussed and resolved through team discussion.

Two independent authors (YB and LYH) extracted data using established forms. Items extracted included study characteristics (authors, year, study design, and sample size), participants (age, sex, and stage of disease), interventions (WBV training protocol and duration), comparators, and outcomes (instruments, pre- and post-intervention outcomes). Results reported as figures were retrieved using Getdata software to obtain means and standard deviations (SD). When discrepancies arose between reviewers, a third author (QZS) was consulted to verify the data and facilitate consensus. Due to variations in the methodologies employed to assess physical function across the studies analyzed, a consultation was conducted to categorize them accordingly (Table 1).

**Table 1:** Physical function assessments included in this review

Outcomes	Assessment
Cardiopulmonary fitness	6-min walk test (6MWT), Peak oxygen uptake (VO_2 peak_), Maximal oxygen consumption (VO_2 max_)
Upper limb muscle strength	Handgrip strength (HGS)
Lower limb muscle strength	60-Second Chair Stand Test (CST 60), Knee extensors maximum voluntary isometric contraction (MVIC), Quadriceps muscle strength, Five-time sit-to-stand test (FTSST), 30-Second Chair Stand Test (CST 30), Lower limb explosive force
Mobility	Time up and go test (TUGT)
Balance	Tinetti balance assessment tool, Modified Berg Scale, Static Balance Tests, Single-Leg Stand Test, and Balance platform

### Risk of bias assessment

Two authors (YB and FZ) evaluated the included studies independently. The Risk Of Bias In Non-randomized Studies-of Interventions (ROBINS-I) [19] tool was used to assess the risk of bias in non-RCTs, while the Cochrane revised tool (ROB-2) was utilized for RCTs [20]. Any inconsistencies were resolved through consensus with the review team.

### Data synthesis and statistical analysis

The meta-analysis of the studies under consideration was conducted utilizing the *meta* [21] package in R software. The analysis involved the comparison of within-group differences and between-group differences, with the mean/SD and sample size for the WBV group of the RCT, and the pre- (baseline) and post-intervention (endpoint) for the single-arm trial being extracted for the first comparison. The second comparison involved pooling the pre-and post-intervention differences between the WBV group and control group of the RCT. The overall effect was assessed using a z-statistic with a *P* value of 0.05. Standardized mean difference (SMD) with a 95% confidence interval (95% CI) is used as summary statistics when evaluation methods are inconsistent; otherwise, the mean difference (MD) is chosen. Furthermore, 95% prediction intervals (95% PI) were calculated to forecast the actual effects' range [22].

The heterogeneity of studies was determined by the χ^2^ statistic and quantified by the *I*^2^ statistic, with Chi-squared tests of *P* < 0.10 and *I*^2^ > 50% indicating the presence of heterogeneity [23]. The Hartung-Knapp-Sidik-Jonkman (HKSJ) method was used as an estimator for the random effects meta-analysis because it consistently has a lower error rate than the DerSimonian-Laird method, particularly when the number of studies is small [24]. If for low heterogeneity, we pool the results using a fixed-effects model because, in this case, the fixed-effects approach outperforms the HKSJ in terms of type I errors [24].

Due to the limited number of included studies (< 10 per variable), meta-regression and funnel plot asymmetry tests were not performed [25]. The robustness of the results was assessed using the "leave-one-out" sensitivity.

### The overall quality of the evidence

Two independent reviewers (YB and LYH) assessed the cumulative evidence for each outcome using the Grading of Recommendations, Assessment, Development, and Evaluation (GRADE) framework [26]. Considering the limited number of between-group differences in studies, we only rated the evidence for within-group differences in outcomes.

## Results

### Study selection

**Fig. 1:**
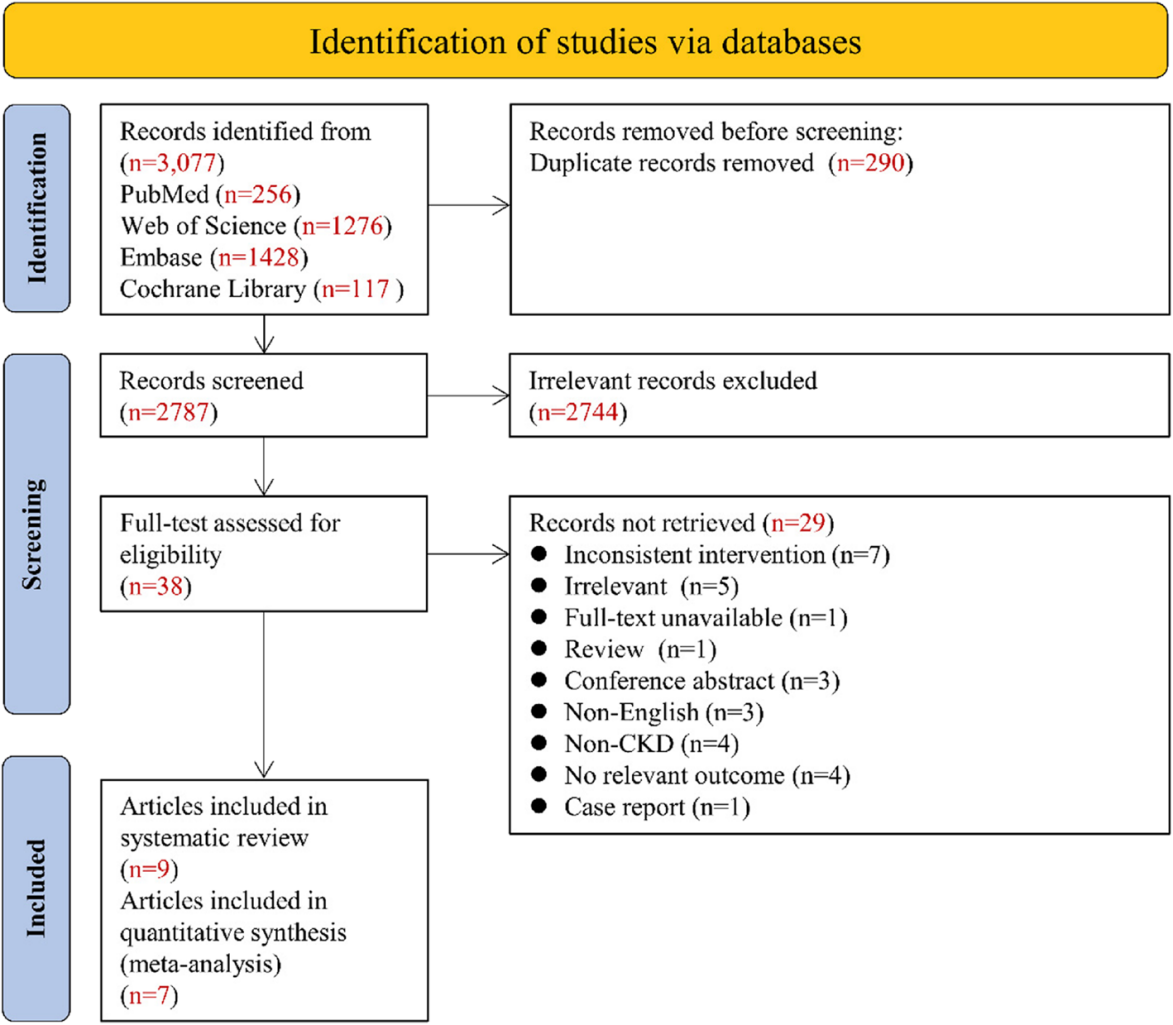
Flow chart of literature screening

The search strategy identified 3,077 records. Figure 1 displays the entire flowchart of the study screening process. A full-text assessment excluded 29 papers, and the remaining nine studies (five RCTs and four single-arm trials) were included in this review [27–35]. Two studies with incomplete data reports and emails sent to obtain unsuccessful, not included in the meta-analysis [27, 31]. The reasons for exclusion are shown in Table S4.

### Study characteristics and quality assessment

Table S5 presents the characteristics of the studies included in this systematic review. The pooled sample consisted of 237 participants (139 males and 98 females), with each study having a range of 5 to 98 participants. Six studies recruited patients with hemodialysis-dependent CKD, while three included kidney transplant recipients. The ages of the participants ranged from 43.7 to 76 years. Table S6 summarizes the risk of bias for the included studies.

### Effect of WBV therapy on physical function in CKD patients (Within-group differences)

#### Cardiopulmonary fitness

**Fig. 2:**
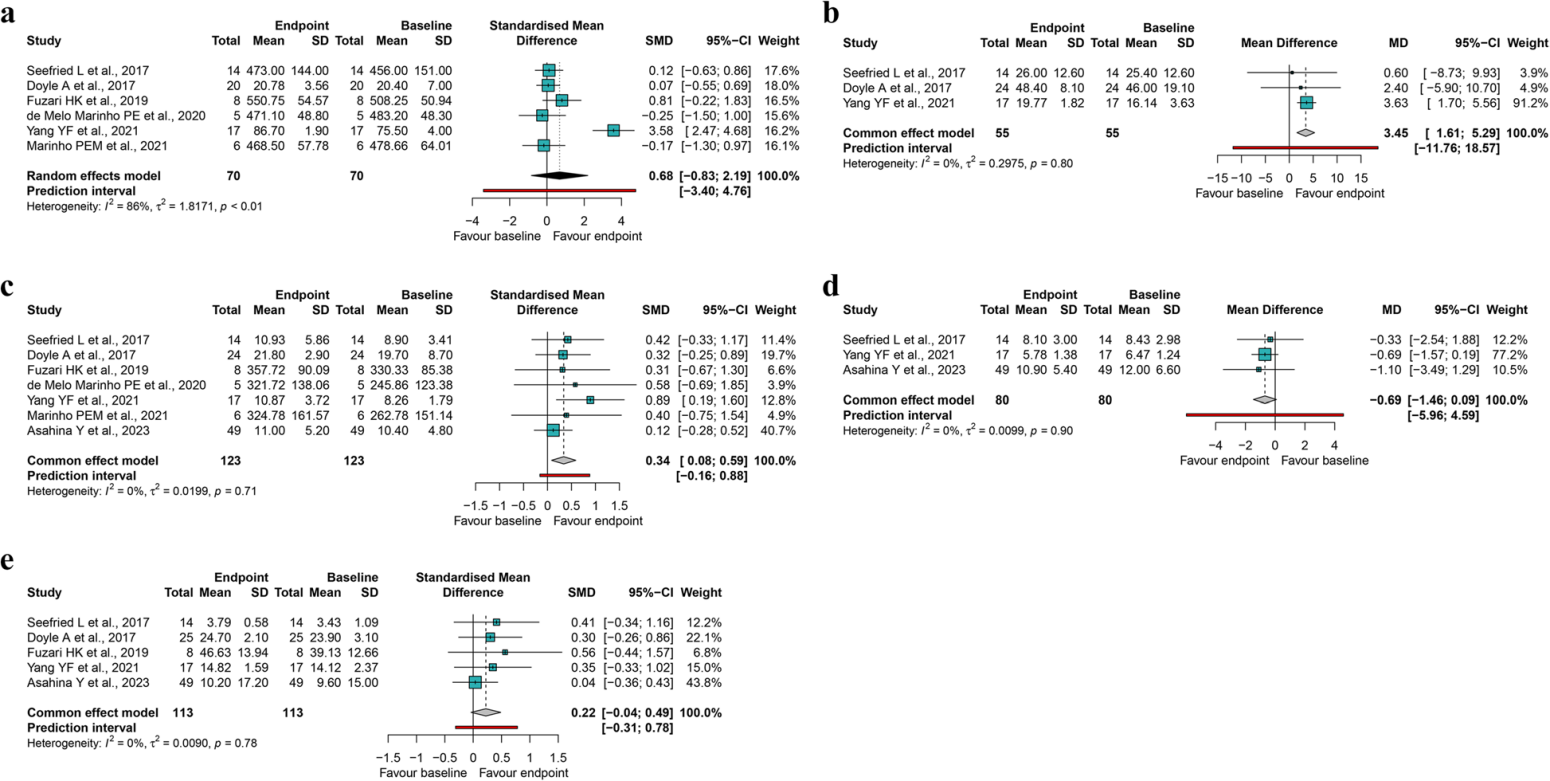
Forest plot and pooled estimates of the impact of WBV training on physical function as compared to baseline. **a** Cardiopulmonary fitness; **b** Upper limb muscle strength; **c** Lower limb muscle strength; **d** Mobility; **e** Balance. Abbreviations: SMD, standardized mean difference; 95% CI, 95% confidence intervals

Seven trials reported cardiopulmonary fitness outcomes, and one study had incomplete data [31], so six trials measuring cardiopulmonary fitness in 70 patients were included in the meta-analysis (Fig. 2a). Four trials used the 6MWT, one used the VO2 peak, and one used the 2-min walk test to substitute the 6MWT. Compared to baseline, WBV treatment resulted in 0.68 SMD (95% CI -0.83 to 2.19) units higher endpoint values for cardiopulmonary fitness, with considerable heterogeneity (*I*^2^ = 86%, *P* < 0.01). 95% PI values ranged from -3.40 to 4.76, suggesting that WBV treatment may not improve cardiopulmonary fitness compared to baseline in future studies in similar settings.

### Lower limb muscle strength

Three trials measuring upper limb muscle strength (assessed by HGS) in 55 patients were pooled in the meta-analysis (Fig. 2b). Compared to baseline, WBV treatment improved HGS by approximately 3.45 kg (95% CI 1.61 to 5.29) in patients with CKD, with no detectable heterogeneity (*I*^2^ = 0%, *P* = 0.80). 95% PI values ranged from -11.76 to 18.57, suggesting that WBV treatment may not significantly improve handgrip strength relative to baseline in future studies.

### Upper limb muscle strength

Eight studies reported lower limb strength, and data from Fuzari HKB et al. [27] could not be transformed. A meta-analysis of seven trials of lower limb muscle strength in 123 patients was performed (Fig. 2c). Four trials used the Chair Stand Test, two used quadriceps muscle strength, and one used knee extensors maximum voluntary isometric contraction. Compared to baseline, WBV treatment significantly increased lower limb muscle strength in patients with CKD (SMD = 0.34, 95% CI 0.08 to 0.59), and no heterogeneity was observed (*I*^2^ = 0%, *P* = 0.71). 95% PI values ranged from -0.16 to 0.88, suggesting that WBV therapy may not significantly improve lower extremity muscle strength compared to baseline in future studies conducted under similar conditions.

### Mobility

A meta-analysis of three trials measuring mobility (assessed by TUGT) in 80 patients was performed (Fig. 2d). WBV therapy shortened the TUGT performance in patients with CKD by approximately 0.69 s (95% CI -1.46 to 0.09) compared to baseline, with no heterogeneity (*I*^2^ = 0%, *P* = 0.90). 95% PI values ranged from -5.96 to 4.59, suggesting that WBV therapy performed in future studies may not improve TUGT performance.

### Balance

A meta-analysis of five trials measuring balance function in 113 patients was conducted (Fig. 2e). Two trials used a balance-specific scale (Tinetti balance assessment tool and Modified Berg Scale), two trials used a balance function test (Static Balance Tests and Single-Leg Stand Test), and one study drew on a balance platform. Compared to baseline, WBV treatment improved balance by 0.22 SMD units (95% CI -0.04 to 0.49) in patients with CKD with no heterogeneity (*I*^2^ = 0%, *P* = 0.78). 95% PI values ranged from -0.31 to 0.78, suggesting that WBV therapy may not improve balance compared to baseline in future studies conducted under similar conditions.

### Sensitivity analysis

Sensitivity analysis using "leave-one-out" did not significantly change the above results (Figure S1).

### Effect of WBV therapy on physical function in CKD patients (Between-group differences)

Pooled results of baseline-endpoint differences from three RCTs (one study with incomplete data was not included) suggest that WBV treatment may tend to improve lower extremity muscle strength, cardiorespiratory fitness, and balance function compared to controls. However, none were statistically significant (Figure S2).

### Summary of quality of evidence

A summary of the results of a meta-analysis of within-group differences in the overall quality of evidence for the GRADE assessment is shown in Table S7. Due to a potential bias in the single-arm test, insufficient sample size, and overly broad confidence intervals, the evidence for cardiorespiratory fitness, mobility, and balance was rated as very low, while both muscle strength results crossed the null line and were scored as low-level evidence.

### Studies included in the qualitative review

The results of an RCT by Maia TO et al. [31] incorporating 12 kidney transplant recipients showed that 12 weeks of WBV therapy did not alter the VO 2max of the subjects. An RCT by Fuzari HKB et al. [27] enrolling 14 hemodialysis-dependent CKD patients with a 12-week WBV treatment in the intervention group and a sham intervention in the control group showed that WBV training attenuated lower extremity explosive strength loss in CKD patients.

For quality-of-life outcomes, included studies reported incompletely or had little data; therefore, no meta-analysis was performed. A single-arm trial with a small sample (n = 5) reported improved pain, social aspects, and mental health scores [32]. The study's results by Doyle et al. [23] showed improvements in the symptoms listed and overall health domains of the Kidney Disease Quality of Life scale after WBV training. The studies by Fuzari HK et al. [34] and Yang YF et al. [33] showed no significant changes in quality-of-life scores for either endpoint.

### Adverse event

Merely two studies incorporated adverse event registration. In the study by Doyle A et al. [29], one patient experienced nausea after using the vibration equipment, while Asahina Y et al. [35] did not report any serious adverse events throughout the study.

## Discussion

### Main findings of the present study

This systematic review and meta-analysis evaluated the effect of WBV training on physical function and quality of life in patients with CKD. The analysis combined nine studies (seven in the meta-analysis) that included 237 participants, mainly hemodialysis-dependent CKD patients and kidney transplant recipients. Pooled data suggest that WBV is an effective training modality for improving muscle strength (both upper and lower extremities) in CKD patients (low evidence); despite a lack of statistical significance, it tends to improve cardiorespiratory fitness, mobility, and balance function. Feasibility was also ensured by further observation of adverse events. The results of this study provide a rationale for WBV-based renal rehabilitation.

### Comparison with other studies

In the non-CKD population, a meta-analysis [36] that included nine studies showed that WBV significantly improved motor function and walking stability in patients with Parkinson's disease but did not have statistically significant improvements in balance. Our meta-analysis found a beneficial effect of WBV on lower limb strength in the CKD population, similar to the results of Zhao Q et al. [37], whose network meta-analysis showed that WBV training was associated with improved knee extension strength (MD: 6.46; 95% CI 1.71 to 11.20) in end-stage renal disease patients.

In another study, Gonçalves de Oliveira R et al. [38] also reported that WBV effectively enhanced lower limb muscle strength in older adults, but no significant effect was observed in the upper limb. The authors likewise concluded that more research is needed to understand the impact of WBV on human physiology. As for safety issues, the systematic review by Coelho-Oliveira et al. [17] reported that low-intensity WBV interventions were well tolerated in patients with CKD with no adverse effects.

Unfortunately, the impact of WBV on the quality of life in CKD patients is still debatable due to the limited number of studies reported. Nevertheless, it is foreseeable that improvements in lower limb muscle function with WBV favor individual quality of life, a hypothesis confirmed in a study that recruited a small sample of patients with multiple sclerosis [39]. Overall, WBV has the potential to improve quality of life, particularly for individuals seeking to enhance physical fitness, manage certain medical conditions, or reduce the risk of falls and injuries [40, 41]. However, its efficacy and safety should be discussed with a healthcare provider before incorporating it into a routine.

### Implication and explanation of findings

Cardiorespiratory fitness is a well-established indicator to assess cardiovascular health status and directly reflects the exercise capacity of an individual [42]. In patients with CKD, cardiorespiratory fitness decreases with worsening renal function [43] and is independently associated with higher mortality [44]. Summary analysis suggests that WBV training may have potential benefits in improving cardiorespiratory fitness in patients with CKD; however, lack of statistical level support, limited data, and potential risk of bias reduce the credibility of the study results. Importantly, this suggests the need for future methodologically robust trials.

Muscle strength is another critical dimension of physical function that affects the prognosis of CKD [45]. Pooled analysis of a single group before and after the intervention showed significant improvements in upper and lower extremity muscle strength. A combined MD in HGS was 3.45 kg higher than baseline, which is clinically meaningful across different types of chronic disease. Our prior systematic review and meta-analysis showed that each unit increase in HGS in CKD patients was associated with a 3.9% reduction in mortality risk (hazard ratio: 0.961; 95% CI 0.949 to 0.974) [46]. Lower limb muscle strength is even more critical for CKD patients to participate in activities of daily living [47]. Nevertheless, pooled single-arm trials did not have a comparison group, so the risk of bias in the results is high, and this finding must be interpreted with caution. Furthermore, including different outcome measures led to the use of SMD in the meta-analysis of lower limb muscle strength, and we could not infer the clinical significance of the specific SMD results.

Better mobility and balance are crucial to preventing falls in patients with CKD [48]. None of the meta-analyses combining a few clinical studies observed statistical significance, but the results were favored towards the endpoints. Possible reasons for the significant improvement in lower extremity muscle strength, while mobility and balance have not yet changed significantly, include 1) the relatively small number of patients included in this analysis, 2) potential bias from inconsistent measurements, and 3) the low frequency of WBV interventions, which may not be observable in the short term. A recent network meta-analysis that included 25 RCTs suggests that high-frequency WBV may be the best option for improving balance in older adults [49].

### Future direction

The application of WBV training continues to evolve, and its clinical value has emerged prominently. However, kidney disease is often intertwined with multiple etiologies, and precise renal rehabilitation requires additional research evidence to support it. This systematic review confirms that the potential health benefits of WBV training greatly outweigh the effects of adverse events, with improved physical function outcomes found in patients with CKD. Although the included RCTs were all low-risk, four points worth noting in future studies are 1) a larger sample size should have been recruited; 2) analysis of the long-term effects of WBV training; and 3) whether adverse events occur at higher magnitudes; 4) focus on the impact of WBV training on quality of life. Finally, future RCTs should still adhere to the Consolidated Standards of Reporting Trials statement to improve the quality of reporting.

### Strengths and limitations

This study is the first meta-analysis of published clinical trials examining the role of WBV training on physical function in patients with CKD. However, there are some limitations to this review. First, the small sample size of the included studies and the potential risk of bias in the included single-arm trials reduce the certainty of the evidence. Second, the practicality of WBV training is somewhat restricted by the diverse parameters and protocols of the intervention, including frequency, amplitude, position of individuals on the platform, and type of platform. Third, the inclusion of solely hemodialysis-dependent CKD patients and kidney transplant recipients in this study may restrict the generalizability of the findings to other stages of CKD populations. Fourth, this meta-analysis was not tested for publication bias due to the limitations of the included studies, but several of the included studies may have had a small sample bias that exaggerated the existing findings. Finally, while using SMD enables the comparison of multiple outcome measures on varying tools, its application has limitations. The SD of a measure is prone to variation across populations, impeding the generalizability of SMD and potentially confounding observations in meta-analyses.

## Conclusion

Utilizing WBV as a rehabilitation training strategy appears to be a viable and pragmatic approach to improving physical function, precisely muscle strength, in individuals diagnosed with CKD.

### Supplementary Information

**Additional file 1:** **Table S1.** The PRISMA 2020 Checklist. **Table S2.** Protocol deviations. **Table S3.** Search detailed for database. **Table S4.** List of studies excluded at full-text review and reasons for exclusion. **Table S5.** Characteristic of the included studies. **Table S6.** Risk of bias for including studies. **Table S7.** GRADE evidence profile for overall quality of evidence assessment. **Figure S1.** Sensitivity analysis for within-group differences. **Figure S2.** Effect of WBV therapy on physical function in CKD patients (Between-group differences).

## Data Availability

The data underlying this article will be shared on reasonable request to the corresponding author.
