# Supplementary material for: Effects of whole-body vibration exercise on physical function in patients with chronic kidney disease: a systematic review and meta-analysis
Source: BMC Nephrol. 2024 Jan 3;25:2. doi: 10.1186/s12882-023-03436-3 (PMC10763333; doi:10.1186/s12882-023-03436-3)
Supplement: Supplementary file 1 — Additional file 1: Table S1. The PRISMA 2020 Checklist. Table S2. Protocol deviations. Table S3. Search detailed for database. Table S4. List of studies excluded at full-text review and reasons for exclusion. Table S5. Characteristic of the included studies. Table S6. Risk of bias for including studies. Table S7. GRADE evidence profile for overall quality of evidence assessment. Figure S1. Sensitivity analysis for within-group differences. Figure S2. Effect of WBV therapy on physical function in CKD patients (Between-group differences). [file 12882_2023_3436_MOESM1_ESM.docx]

**Effects of whole-body vibration exercise on physical function in patients with chronic kidney disease: a systematic review and meta-analysis**

**Supplementary Material**

**Content**

[Table S1 The PRISMA 2020 Checklist 2](#_Toc138013454)

[Table S2 Protocol deviations. 7](#_Toc138013455)

[Table S3 Search detailed for database. 8](#_Toc138013456)

[Table S4 List of studies excluded at full-text review and reasons for exclusion. 11](#_Toc138013457)

[Table S5 Characteristic of the included studies 14](#_Toc138013458)

[Table S6 Risk of bias for including studies. 18](#_Toc138013459)

[Table S7 GRADE evidence profile for overall quality of evidence assessment 20](#_Toc138013460)

[Figure S1 Sensitivity analysis for within-group differences 23](#_Toc138013461)

[Figure S2 Effect of WBV therapy on physical function in CKD patients (Between-group differences). 24](#_Toc138013462)

[Reference 25](#_Toc138013463)

Table S1 The PRISMA 2020 Checklist

| **Section and Topic** | **Item #** | **Checklist item** | **Location where item is reported** |
| --- | --- | --- | --- |
| **TITLE** | | |  |
| Title | 1 | Identify the report as a systematic review. | Title page |
| **ABSTRACT** | | |  |
| Abstract | 2 | See the PRISMA 2020 for Abstracts checklist. | Abstract |
| **INTRODUCTION** | | |  |
| Rationale | 3 | Describe the rationale for the review in the context of existing knowledge. | Page 5 |
| Objectives | 4 | Provide an explicit statement of the objective(s) or question(s) the review addresses. | Page 5-6 |
| **METHODS** | | |  |
| Eligibility criteria | 5 | Specify the inclusion and exclusion criteria for the review and how studies were grouped for the syntheses. | Page 7 |
| Information sources | 6 | Specify all databases, registers, websites, organisations, reference lists and other sources searched or consulted to identify studies. Specify the date when each source was last searched or consulted. | Page 6-7 |
| Search strategy | 7 | Present the full search strategies for all databases, registers and websites, including any filters and limits used. | Table S3 |
| Selection process | 8 | Specify the methods used to decide whether a study met the inclusion criteria of the review, including how many reviewers screened each record and each report retrieved, whether they worked independently, and if applicable, details of automation tools used in the process. | Page 7 |
| Data collection process | 9 | Specify the methods used to collect data from reports, including how many reviewers collected data from each report, whether they worked independently, any processes for obtaining or confirming data from study investigators, and if applicable, details of automation tools used in the process. | Page 7 and Table 1 |
| Data items | 10a | List and define all outcomes for which data were sought. Specify whether all results that were compatible with each outcome domain in each study were sought (e.g. for all measures, time points, analyses), and if not, the methods used to decide which results to collect. | Page 7 and Table 1 |
|  | 10b | List and define all other variables for which data were sought (e.g. participant and intervention characteristics, funding sources). Describe any assumptions made about any missing or unclear information. | Table 1 |
| Study risk of bias assessment | 11 | Specify the methods used to assess risk of bias in the included studies, including details of the tool(s) used, how many reviewers assessed each study and whether they worked independently, and if applicable, details of automation tools used in the process. | Page 8 |
| Effect measures | 12 | Specify for each outcome the effect measure(s) (e.g. risk ratio, mean difference) used in the synthesis or presentation of results. | Page 8 |
| Synthesis methods | 13a | Describe the processes used to decide which studies were eligible for each synthesis (e.g. tabulating the study intervention characteristics and comparing against the planned groups for each synthesis (item #5)). | Page 8 |
|  | 13b | Describe any methods required to prepare the data for presentation or synthesis, such as handling of missing summary statistics, or data conversions. | Page 8 |
|  | 13c | Describe any methods used to tabulate or visually display results of individual studies and syntheses. | Page 8 |
|  | 13d | Describe any methods used to synthesize results and provide a rationale for the choice(s). If meta-analysis was performed, describe the model(s), method(s) to identify the presence and extent of statistical heterogeneity, and software package(s) used. | Page 8 |
|  | 13e | Describe any methods used to explore possible causes of heterogeneity among study results (e.g. subgroup analysis, meta-regression). | Page 8 |
|  | 13f | Describe any sensitivity analyses conducted to assess robustness of the synthesized results. | Page 8 |
| Reporting bias assessment | 14 | Describe any methods used to assess risk of bias due to missing results in a synthesis (arising from reporting biases). | Page 8 |
| Certainty assessment | 15 | Describe any methods used to assess certainty (or confidence) in the body of evidence for an outcome. | Page 9 |
| **RESULTS** | | |  |
| Study selection | 16a | Describe the results of the search and selection process, from the number of records identified in the search to the number of studies included in the review, ideally using a flow diagram. | Page 9 and Figure 1 |
|  | 16b | Cite studies that might appear to meet the inclusion criteria, but which were excluded, and explain why they were excluded. | Table S4 |
| Study characteristics | 17 | Cite each included study and present its characteristics. | Table S5 |
| Risk of bias in studies | 18 | Present assessments of risk of bias for each included study. | Table S6 |
| Results of individual studies | 19 | For all outcomes, present, for each study: (a) summary statistics for each group (where appropriate) and (b) an effect estimate and its precision (e.g. confidence/credible interval), ideally using structured tables or plots. | Figure 2 |
| Results of syntheses | 20a | For each synthesis, briefly summarise the characteristics and risk of bias among contributing studies. | Page 9-11 |
|  | 20b | Present results of all statistical syntheses conducted. If meta-analysis was done, present for each the summary estimate and its precision (e.g. confidence/credible interval) and measures of statistical heterogeneity. If comparing groups, describe the direction of the effect. | Page 9-11 |
|  | 20c | Present results of all investigations of possible causes of heterogeneity among study results. | None |
|  | 20d | Present results of all sensitivity analyses conducted to assess the robustness of the synthesized results. | Figure S2 |
| Reporting biases | 21 | Present assessments of risk of bias due to missing results (arising from reporting biases) for each synthesis assessed. | Page 10-11 |
| Certainty of evidence | 22 | Present assessments of certainty (or confidence) in the body of evidence for each outcome assessed. | Table S7 |
| **DISCUSSION** | | |  |
| Discussion | 23a | Provide a general interpretation of the results in the context of other evidence. | Page 12-13 |
|  | 23b | Discuss any limitations of the evidence included in the review. | Strengths and limitations |
|  | 23c | Discuss any limitations of the review processes used. | Page 13 |
|  | 23d | Discuss implications of the results for practice, policy, and future research. | Page 12-13 |
| **OTHER INFORMATION** | | |  |
| Registration and protocol | 24a | Provide registration information for the review, including register name and registration number, or state that the review was not registered. | Page 6 |
|  | 24b | Indicate where the review protocol can be accessed, or state that a protocol was not prepared. | Page 6 |
|  | 24c | Describe and explain any amendments to information provided at registration or in the protocol. | Table S2 |
| Support | 25 | Describe sources of financial or non-financial support for the review, and the role of the funders or sponsors in the review. | Page 16 |
| Competing interests | 26 | Declare any competing interests of review authors. | Page 16 |
| Availability of data, code and other materials | 27 | Report which of the following are publicly available and where they can be found: template data collection forms; data extracted from included studies; data used for all analyses; analytic code; any other materials used in the review. | Page 16 |

Table S2 Protocol deviations.

| **Section** | **Previous protocol** | **Publication** |
| --- | --- | --- |
| Author | Yan bai, Fan Zhang, XiaoJing Yin, Qiuzi Sun. | Yan Bai, Liuyan Huang, Xiaojing Yin, Qiizi Sun, Fan Zhang. |
| Literature search | Studies will be searched through PubMed, Web of Science, the Cochrane Library, and Embase databases from inception to April 2023. | Studies was searched through PubMed, Web of Science, the Cochrane Library, and Embase databases from inception to March 2023 and updated in June 2023. |
| Types of study to be included | Randomized controlled trials. | The study design was expanded to include clinical trials, i.e., RCTs, quasi-experimental studies, and single-arm trials. |
| Risk of bias assessment | Joanna Brings Institute Meta-Analysis of Statistics Assessment and Review Instrument. | Version 2 of the Cochrane-RoB tool (RoB-2) and Risk of Bias In Non-randomized Studies-of Interventions (ROBINS-I) |
| Analysis of subgroups or subsets | Subgroup analysis with different vibration frequencies and vibration times. | Due to the limited number of included studies, we were unable to perform subgroup analyses. |

Table S3 Search detailed for database.

| **Databases** | **#** | **Search strategy** |
| --- | --- | --- |
| PubMed | 1 | "renal insufficiency, chronic"[MeSH Terms] |
|  | 2 | "chronic renal insufficiency"[Title/Abstract] or "chronic kidney insufficiency"[Title/Abstract] or "chronic kidney disease"[Title/Abstract] or "chronic renal disease"[Title/Abstract] |
|  | 3 | "CKD"[Title/Abstract] or "CKF"[Title/Abstract] or "CRD"[Title/Abstract] or "CRF"[Title/Abstract] |
|  | 4 | "end-stage kidney"[Title/Abstract] or "end-stage renal"[Title/Abstract] or "end-stage kidney"[Title/Abstract] or "end-stage renal"[Title/Abstract] |
|  | 5 | "ESRD"[Title/Abstract] or "ESRF"[Title/Abstract] or "ESKD"[Title/Abstract] or "ESKF"[Title/Abstract] |
|  | 6 | "Renal Replacement Therapy"[MeSH Terms] |
|  | 7 | "dialysis"[Title/Abstract] |
|  | 8 | "hemodialysis"[Title/Abstract] or "haemodialysis"[Title/Abstract] or "hemodiafiltration"[Title/Abstract] or "haemodiafiltration"[Title/Abstract] or "HD"[Title/Abstract] |
|  | 9 | "PD"[Title/Abstract] |
|  | 10 | "renal transplantation"[Title/Abstract] or "kidney grafting"[Title/Abstract] or "kidney transplantation"[Title/Abstract] |
|  | 11 | "KTRs"[Title/Abstract] |
|  | 12 | or/1-11 |
|  | 13 | "Vibration"[MeSH Terms] |
|  | 14 | "whole body vibration" [Title/Abstract] or "whole-body vibration" [Title/Abstract] or "vibration exercise" [Title/Abstract] or "vibration training" [Title/Abstract] or "WBV" [Title/Abstract] or "vibration therapy" [Title/Abstract] or "constant frequency vibration"[Title/Abstract] or "whole-body sinusoidal vibration"[Title/Abstract] |
|  | 15 | or/13-14 |
|  | 16 | and/12,15 |
| Embase | 1 | 'kidney disease'/exp |
|  | 2 | ('chronic kidney disease' or 'chronic renal disease' or 'chronic kidney failure' or 'chronic renal failure'):ti,ab,kw |
|  | 3 | (CKF or CKD or CRFor CRD):ti,ab,kw |
|  | 4 | ('end-stage kidney' or 'end-stage renal' or 'endstage kidney' or 'endstage renal'):ti,ab,kw |
|  | 5 | (ESRD or ESRF or ESKD or ESKF):ti,ab,kw |
|  | 6 | 'renal replacement therapy'/exp |
|  | 7 | 'dialysis':ti,ab,kw |
|  | 8 | (hemodialysis or haemodialysis or hemofiltration or haemofiltration or hemodiafiltration or haemodiafiltration or HD):ti,ab,kw |
|  | 9 | PD:ti,ab,kw |
|  | 10 | ('renal transplantation' or 'kidney grafting' or 'kidney transplantation'):ti,ab,kw |
|  | 11 | KTRs:ti,ab,kw |
|  | 12 | or/1-11 |
|  | 13 | ' Vibration'/exp |
|  | 14 | ('whole body vibration ' or ' whole-body vibration exercise' or 'vibration exercise' or 'vibration training' or 'WBV' or 'vibration therapy' or 'constant frequency vibration' or 'whole-body sinusoidal vibration'): ti,ab,kw |
|  | 15 | or/13-14 |
|  | 16 | and/12,15 |
| Web of Science | 1 | TS=("chronic kidney disease" or "chronic renal disease" or "chronic kidney failure" or "chronic renal failure" or CKD or CRD or CKF or CRF) |
|  | 2 | TS=("end-stage kidney" or "end-stage renal" or "endstage kidney" or "endstage renal" or ESKD or ESKF or ESRD or ESRF) |
|  | 3 | TS=("renal replacement therapy" or dialysis or hemodialysis or haemodialysis or hemofiltration or hemodiafiltrations or HD or PD) |
|  | 4 | TS=("renal transplantation" or "kidney grafting" or "kidney transplantation" or KTRs) |
|  | 5 | or/1-4 |
|  | 6 | TS=("Vibration" or "whole body vibration" or "whole-body vibration" or "vibration exercise" or "vibration training" or "WBV" or "vibration therapy" or "constant frequency vibration" or "whole-body sinusoidal vibration") |
|  | 7 | and/5,6 |
| Cochrane Library | 1 | "renal insufficiency, chronic"[MeSH Terms] |
|  | 2 | ('chronic renal insufficiency' or 'chronic kidney insufficiency' or 'chronic kidney disease' or ' chronic renal disease'): ti,ab,kw |
|  | 3 | (CKD or CKF or CRD or CRF): ti,ab,kw |
|  | 4 | ('end-stage kidney' or 'end-stage renal' or 'end-stage kidney' or 'end-stage renal'): ti,ab,kw |
|  | 5 | (ESRD or ESRF or ESKD or ESKF or KTRs): ti,ab,kw |
|  | 6 | "Renal Replacement Therapy"[MeSH Terms] |
|  | 7 | ('dialysis "hemodialysis' or 'haemodialysis' or 'hemodiafiltration' or 'haemodiafiltration' or 'HD' or 'PD'): ti,ab,kw |
|  | 8 | ('renal transplantation' or "kidney grafting' or 'kidney transplantation'): ti,ab,kw |
|  | 9 | or/1-8 |
|  | 10 | 'Vibration'[MeSH Terms] |
|  | 11 | ('whole body vibration' or 'whole-body vibration exercise' or 'vibration exercise' or 'vibration training' or 'WBV' or 'vibration therapy' or 'constant frequency vibration' or 'whole-body sinusoidal vibration'): ti,ab,kw |
|  | 12 | or/10-11 |
|  | 13 | and/9,12 |

Table S4 List of studies excluded at full-text review and reasons for exclusion.

| **NO.** | **Reference** | **Exclusion reason** |
| --- | --- | --- |
| 1 | Coelho-Oliveira AC, Silva ABJD, Braga SS, et al. Effects of whole-body vibration exercise in patients with chronic kidney disease: a systematic review. Disabil Rehabil. 2023;45(3):415-424. doi:10.1080/09638288.2022.2037750 | Review |
| 2 | Edwards AE, Kopple JD, Kornfeld CM. Vibrotactile threshold in patients undergoing maintenance hemodialysis. Arch Intern Med. 1973;132(5):706-708. | Non-English |
| 3 | Ossenkop C, Quellhorst E. Einschränkung der Vibrationsempfindung als Parameter zur Früherkennung der nephrogenen Polyneuropathie [Reduction of vibration-perception as a parameter for the early diagnosis of nephrogenic polyneuropathy]. Verh Dtsch Ges Inn Med. 1976;82 Pt 2:1544-1545. | Non-English |
| 4 | Daniel CR, Bower JD, Pearson JE, Holbert RD. Vibrometry and neuropathy. J Miss State Med Assoc. 1977;18(2):30-32. | Full-text unavailable |
| 5 | Werneck LC, Mulinari AS, Laffitte A, Kesikowski LJ. Polineuropatia uremica: estudo do limiar de percepção vibratória em 19 pacientes [Uremic polyneuropathy: a study of vibratory perception threshold in 19 patients]. Arq Neuropsiquiatr. 1984;42(3):215-220. doi:10.1590/s0004-282x1984000300004 | Non-English |
| 6 | Rudoy J, Kohan R, Ben-Ari J. Externally applied abdominal vibration as a method for improving efficiency of peritoneal dialysis. Nephron. 1987;46(4):364-366. doi:10.1159/000184391 | No relevant outcome |
| 7 | Lee PT, Fang HC, Chen CL, Chung HM, Chiou YH, Chou KJ. High vibration perception threshold and autonomic dysfunction in hemodialysis patients with intradialysis hypotension. Kidney Int. 2003;64(3):1089-1094. doi:10.1046/j.1523-1755.2003.00174.x | Inconsistent intervention |
| 8 | Colson SS, Petit PD, Hébreard L, Tessaro J, Pensini M. Whole body vibration does not enhance muscle activation. Int J Sports Med. 2009;30(12):841-844. doi:10.1055/s-0029-1234082 | Non-CKD |
| 9 | Kipp K, Johnson ST, Doeringer JR, Hoffman MA. Spinal reflex excitability and homosynaptic depression after a bout of whole-body vibration. Muscle Nerve. 2011;43(2):259-262. doi:10.1002/mus.21844 | No relevant outcome |
| 10 | Kim JC, Garzotto F, Cruz DN, et al. Enhancement of solute removal in a hollow-fiber hemodialyzer by mechanical vibration. Blood Purif. 2011;31(4):227-234. doi:10.1159/000321073 | Inconsistent intervention |
| 11 | Balal M, Paydas S, Inal M. Unilateral renal venous thrombosis secondary to the use of vibration belt in a young woman. Ren Fail. 2012;34(9):1163-1165. doi:10.3109/0886022X.2012.712848 | Case report |
| 12 | Mueller BA, Jasiak KD, Thiel SR, et al. Vibration enhances clearance of solutes with varying molecular weights during in vitro hemodialysis. ASAIO J. 2013;59(2):140-144. doi:10.1097/MAT.0b013e3182837ff0 | Inconsistent intervention |
| 13 | Colson SS, Petit PD. Lower limbs power and stiffness after whole-body vibration. Int J Sports Med. 2013;34(4):318-323. doi:10.1055/s-0032-1311596 | Non-CKD |
| 14 | Zeigler ZS, Swan PD. Acute effects of whole-body vibration with resistance exercise on postexercise blood pressure and oxygen consumption in prehypertensive adults. J Exerc Sci Fit. 2016;14(1):14-23. doi:10.1016/j.jesf.2015.12.001 | Non-CKD |
| 15 | Lin CH, Kan CD, Chen WL, Wu MJ, Yu FM. An equivalent astable multivibrator model to assess flow instability and dysfunction risk in in-vitro stenotic arteriovenous grafts. Technol Health Care. 2016;24(3):295-308. doi:10.3233/THC-161130 | Irrelevant |
| 16 | Casula EP, Pellicciari MC, Picazio S, Caltagirone C, Koch G. Fronto-parietal changes in oscillatory activity reflects spike-timing-dependent plasticity. Neuroimage. 2016;143:204-213. doi:10.1016/j.neuroimage.2016.08.060 | Conference abstract |
| 17 | Jung JH, Chae YJ, Lee DH, et al. Changes in whole blood viscosity during hemodialysis and mortality in patients with end-stage renal disease. Clin Hemorheol Microcirc. 2017;65(3):285-297. doi:10.3233/CH-16183 | Irrelevant |
| 18 | Fuzari HK, Dornelas de Andrade A, A Rodrigues M, et al. Whole body vibration improves maximum voluntary isometric contraction of knee extensors in patients with chronic kidney disease: A randomized controlled trial. Physiother Theory Pract. 2019;35(5):409-418. doi:10.1080/09593985.2018.1443537 | Conference abstract |
| 19 | YALÇIN B, ERDOGANOGLU Y, KÜLAH E, et al. Assessment of the effect of foot soles on static balance and physical performance in hemodialysis patients: Pilot study[J]. Fizyoterapi Rehabilitasyon, 2018,29(2): S43-S44. | Irrelevant |
| 20 | Fonseca DA, Antunes PE, Antunes MJ, Cotrim MD. Vasomotion as an oscillatory sign of functional impairment in the human internal thoracic artery: A study based on risk factors and vessel reactivity. Exp Physiol.2018;103(7):1030-1038. doi:10.1113/EP087002 | Inconsistent intervention |
| 21 | DEDHIA P, JASANI R H, BILLA V, et al. Vibration perception threshold as a measure of peripheral neuropathy in dialysis patients: A pilot project[J]. Journal of the American Society of Nephrology, 2018,29: 199. | Inconsistent intervention |
| 22 | Ferdousi M, Azmi S, Kalteniece A, et al. No evidence of improvement in neuropathy after renal transplantation in patients with end stage kidney disease. J Peripher Nerv Syst. 2021;26(3):269-275. doi:10.1111/jns.12456 | Irrelevant |
| 23 | SOUZA H D, PESSOA M, CLEMENTE R, et al. Inspiratory muscle training associated with whole body vibration improves diaphragm thickness and mobility but does not affect serum BDNF and peripheral muscle in prefrail older: a randomized double-blind clinical trial[J]. 2019,54. | Non-CKD |
| 24 | SEEFRIED L, GENEST F, LUKSCHE N, et al. Whole body vibration in hemodialysis patients – beneficial effects particularly in patients with impaired physical functions[J]. Pravention und Rehabilitation, 2019,31(4): 156-166. | Conference abstract |
| 25 | TAO X, THWIN O, PRECIADO P, et al. High-frequency oscillations of intradialytic arterial oxygen saturation in hemodialysis patients[J]. Journal of the American Society of Nephrology, 2020,31: 377. | Irrelevant |
| 26 | Santos LMM, Figueiredo PHS, Silva ACR, et al. Determining factors of functioning in hemodialysis patients using the international classification of functioning, disability and health. BMC Nephrol. 2022;23(1):119. Published 2022 Mar 24. doi:10.1186/s12882-022-02719-5 | Inconsistent intervention |
| 27 | Cunningham PS, Kitchen GB, Jackson C, et al. ClinCirc identifies alterations of the circadian peripheral oscillator in critical care patients. J Clin Invest. 2023;133(4):e162775. Published 2023 Feb 15. doi:10.1172/JCI162775 | Inconsistent intervention |
| 28 | Rajapakse CS, Leonard MB, Kobe EA, et al. The Efficacy of Low-intensity Vibration to Improve Bone Health in Patients with End-stage Renal Disease Is Highly Dependent on Compliance and Muscle Response. Acad Radiol. 2017;24(11):1332-1342. doi:10.1016/j.acra.2017.05.014 | No relevant outcome |
| 29 | Hornik B, Duława J, Marcisz C, etal. The Effect of Mechanically-Generated Vibrations on the Efficacy of Hemodialysis; Assessment of Patients' Safety: Preliminary Reports. Int J Environ Res Public Health. 2019;16(4):594. Published 2019 Feb 18. doi:10.3390/ijerph16040594 | No relevant outcome |

Table S5 Characteristic of the included studies

| **Study (Year)** | **Study**  **design** | **Sample, n** | **Age, year** | **Intervention group** | **Control group** | **Duration** | **Outcome Measure** |
| --- | --- | --- | --- | --- | --- | --- | --- |
| Seefried L (2017) [1] | Single arm | 14 hemodialysis patients (8 male/6 female) | 59±11 | **Equipment:** a side-alternating vibratory platform Galileo.  **Frequency:** increased every 4 weeks form initially 5-9 Hz.  **Amplitudes:**  “easy” group: 0.5-1.5 mm  “intermediate” group: 1.5-2.5 mm  “hard” group: 2-3 mm.  **Time:** each exercise phase of 30-60 s was followed by 1-2 min of recovery time. | Not applicable. | 12 weeks | - Static Balance Tests - Timed-up and go test - Five-time sit-to-stand test - Handgrip test - 6-minute walk test |
| Doyle A (2017) [2] | Single arm | 49 hemodialysis patients (27 male/22 female) | 65 | **Frequency:** 50 Hz.  **Amplitudes:** 10 mm.  **Time:** 8 weeks of WBV, thrice weekly for 3min, 3 sessions of 3 min/week. | Not applicable. | 8 weeks | - Tinetti balance assessment - 60-Second Chair Stand Test - Handgrip strength - Peak oxygen uptake - Quality of life |
| Fuzari HKB (2018) [3] | RCT | 14 ESRD or hemodialysis patients (10 male/4 female) | WBV: 62.42±10.32  Con: 53.28±8.26 | **Equipment:** a vibrating platform with vertical vibration.  **Frequency:** 35 Hz fixed.  **Amplitude:** 2 mm and/or 4 mm. | Sham intervention. | 12 weeks | - Lower limb explosive power |
| Fuzari HK (2018) [4] | RCT | 16 ESRD or hemodialysis patients (11 male/ 5 female) | WBV: 61.50±9.91  Con: 53.63±7.70 | **Equipment:** the patient stood on the vibrating platform and remained in a semi-squat static position (30 knee flexion; measured with goniometer) during vibration period, and the upper limb contralateral to the arteriovenous fistula, slightly flexed while supporting on the platform and with feet 20 cm apart. During rest the patients were able to extend the knees to relax.  **Frequency:** 35 Hz fixed frequency.  **Amplitude:** 2-4 mm.  **Time:** 2 times a week for 12 weeks, sessions lasting about 1 h. | Sham intervention. | 12 weeks | - Knee extensors maximum voluntary isometric contraction - 6-minute walk test - Dynamic balance - Quality of life |
| Maia TO (2020) [5] | RCT | 12 kidney transplant recipients (8 male/4 female) | Median 45 | **Equipment:** vibratory platform.  **Frequency:** 35 Hz fixed frequency.  **Time:** 2 times a week. | Sham intervention. | 12 weeks | - Maximal oxygen consumption |
| de Melo Marinho PÉ (2020) [6] | Single arm | 5 kidney transplant recipients (3 male/2 female) | 47.6±4.0 | **Equipment:** synchronous WBV was conducted with the subject standing and stationery, feet 20 cm apart in a semi-squat position and upper limbs slightly flexed and holding on to the platform.  **Frequency:** 35 Hz.  **Amplitude:** 2-4 mm .  **Time:** the vibration duration was 60 s interspersed with 30 s of rest, and the total training time per session progressed from 10 min in the first month, 15 min in the second month and 20 min in the third month. | Not applicable. | 12 weeks | - Quadriceps muscle strength - 6-minute walk test - Quality of life |
| Yang YF (2021) [7] | Single arm | 17 hemodialysis patients (9 male/8 female) | 47.80±9.30 | **Equipment:** the patients stood on a vertical vibration platform to perform WBV exercise.  **Frequency:** 30-50 Hz.  **Amplitude:** 1.5 mm.  **Time:** 30 min per session, thrice a week for 12 weeks; Participants performed squats, deep squats, wide-stance squats, toe stands, 1-legged squats, and lunges. Each exercise posture lasted 3 repetitions from 30 to 45 s with a resting interval of 20-30 s. After WBV exercise, 5-10 min of static stretching exercises was performed for cooling down. | Not applicable. | 12 weeks | - Five times sit-to-stand test - Handgrip strength - 2-minute walk test - Modified Berg balance scale - Quality of life |
| Marinho PEM (2021) [8] | RCT | 12 kidney transplant recipients (8 male/4 female) | WBV: 43.17 (31.16-55.18)  Con: 44.50 (35.38-53.62) | **Equipment:** on the vibrating platform, the subject standing stationary, feet 20 cm apart in a semi-squatting position and upper limbs slightly flexed.  **Frequency:** 35 Hz.  **Amplitude:** 2 mm.  **Time:** twice a week for 12 weeks,each session consisted of 5-10 minutes of stretching for muscle groups of the upper and lower limbs prior to WBV training. | Sham intervention. | 12 weeks | - Quadriceps muscle strength - 6-minute walk test |
| Asahina Y (2023) [9] | RCT | 98 hemodialysis patients (55 male/43 female) | 76±7 | **Instrument:** a vibrating platform.  **Frequency:** 18 Hz during the first week and was increased by 2 Hz/wk to a maximum of 26 Hz.  **Amplitude:** 2.0-3.0 mm.  **Time:** the participants stood on the platform for 3 min with bent knees. | Standard care. | 12 weeks | - Single-leg stand test - 30-second Chair Stand Test - Timed Up and Go Test |

Table S6 Risk of bias for including studies.

| Study | ROBINS-I (Non-Randomized Studies) | | | | | | | |
| --- | --- | --- | --- | --- | --- | --- | --- | --- |
|  | Confounding | Selection bias | Bias in measurement classification of intervention | Bias due to deviations intended interventions | Bias due to missing outcome data | Bias in measurement of outcomes | Bias in selection of reported results | Overall bias |
| Seefried L(2017) | PY | N | Y | N | N | Y | N | Moderate |
| Doyle A (2017) | PY | N | PY | N | Y | Y | N | Moderate |
| de Melo Marinho PÉ (2020) | PN | N | Y | N | N | Y | N | Moderate |
| Yang YF (2021) | PY | N | Y | N | N | Y | N | Moderate |

**Note:** The response options regarding biases included Yes (Y), Probably Yes (PY), Probably No (PN), No (N) and No Information (NI). "Y" indicated a low risk of bias, "PY" indicated a moderate risk of bias, "PN" indicated a serious risk of bias, "N" indicated a critical risk of bias and "NI" indicated that was no information related to bias.

| Study | RoB-2 (randomized controlled trial) | | | | | |
| --- | --- | --- | --- | --- | --- | --- |
|  | Randomization process | Deviations from the intended interventions | Missing outcome data | Measurement of the outcome | Selection of the reported result | Overall bias |
| Fuzari HK (2018) | Low | Low | Low | Low | Low | Low |
| Maia TO (2020) | Low | Low | Low | Low | Low | Low |
| Marinho PEM (2021) | Low | Low | Low | Low | Low | Low |
| Asahina Y (2023) | Low | Low | Low | Low | Low | Low |
| Fuzari HKB (2018) | Low | Low | Low | Low | Low | Low |

Table S7 GRADE evidence profile for overall quality of evidence assessment

| **Certainty assessment** | | | | | | | **№ of patients** | **Effect** | **Certainty** | **Importance** |
| --- | --- | --- | --- | --- | --- | --- | --- | --- | --- | --- |
| **№ of studies** | **Study design** | **Risk of bias** | **Inconsistency** | **Indirectness** | **Imprecision** | **Other considerations** | **[WBV group]** | **Absolute (95% CI)** |  |  |
| **Cardiopulmonary fitness** | | | | | | | | | | |
| 6 | Single arm and RCT | Serious ^a^ | Very serious ^b^ | Not serious ^c^ | Serious ^d^ | 1) Small sample size  2) Dose-response effect | 70 | SMD: 0.68 (-0.83 to 2.19) | ⨁◯◯◯  Very low | Important |
| **Lower limbs muscle strength** | | | | | | | | | | |
| 3 | Single arm and RCT | Serious ^a^ | Not serious ^e^ | Not serious ^c^ | Not serious ^f^ | 1) Small sample size  2) Dose-response effect | 55 | MD: 3.45 (1.61 to 5.29) | ⨁⨁◯◯  Low | Important |
| **Upper limb muscle strength** | | | | | | | | | | |
| 6 | Single arm and RCT | Serious ^a^ | Not serious ^e^ | Not serious ^c^ | Not serious ^f^ | 1) Small sample size  2) Dose-response effect | 123 | SMD: 0.34 (0.08 to 0.59) | ⨁⨁◯◯  Low | Important |
| **Mobility** | | | | | | | | | | |
| 3 | Single arm and RCT | Serious ^a^ | Not serious ^e^ | Not serious ^c^ | Serious ^d^ | 1) Small sample size  2) Dose-response effect | 80 | MD: -0.69 (-1.46 to 0.09) | ⨁◯◯◯  Very low | Important |
| **Balance** | | | | | | | | | | |
| 5 | Single arm and RCT | Serious ^a^ | Not serious ^e^ | Not serious ^c^ | Serious ^d^ | 1) Small sample size  2) Dose-response effect | 113 | SMD: 0.22 (-0.04 to 0.49) | ⨁◯◯◯  Very low | Important |
| RCT: randomized control trial; WBV: whole body vibration; CI: Confidence interval; SMD: Standardized mean difference; MD: Mean difference. | | | | | | | | | | |
| **Explanations**  a. Potential risk of bias in single-arm trials  b. *I*^2^ = 86% > 75%  c. All patients had chronic kidney disease and were receiving only whole-body vibration training or sham interventions.  d. 95% CI excludes line of null effect.  e. *I*^2^ = 0%.  f. 95% CI crosses the line of null effect. | | | | | | | | | | |

Figure S1 Sensitivity analysis for within-group differences





***Note:*** a) Cardiopulmonary fitness; b) Upper limb muscle strength; c) Lower limb muscle strength; d) Mobility; e) Balance.

Figure S2 Effect of WBV therapy on physical function in CKD patients (Between-group differences).


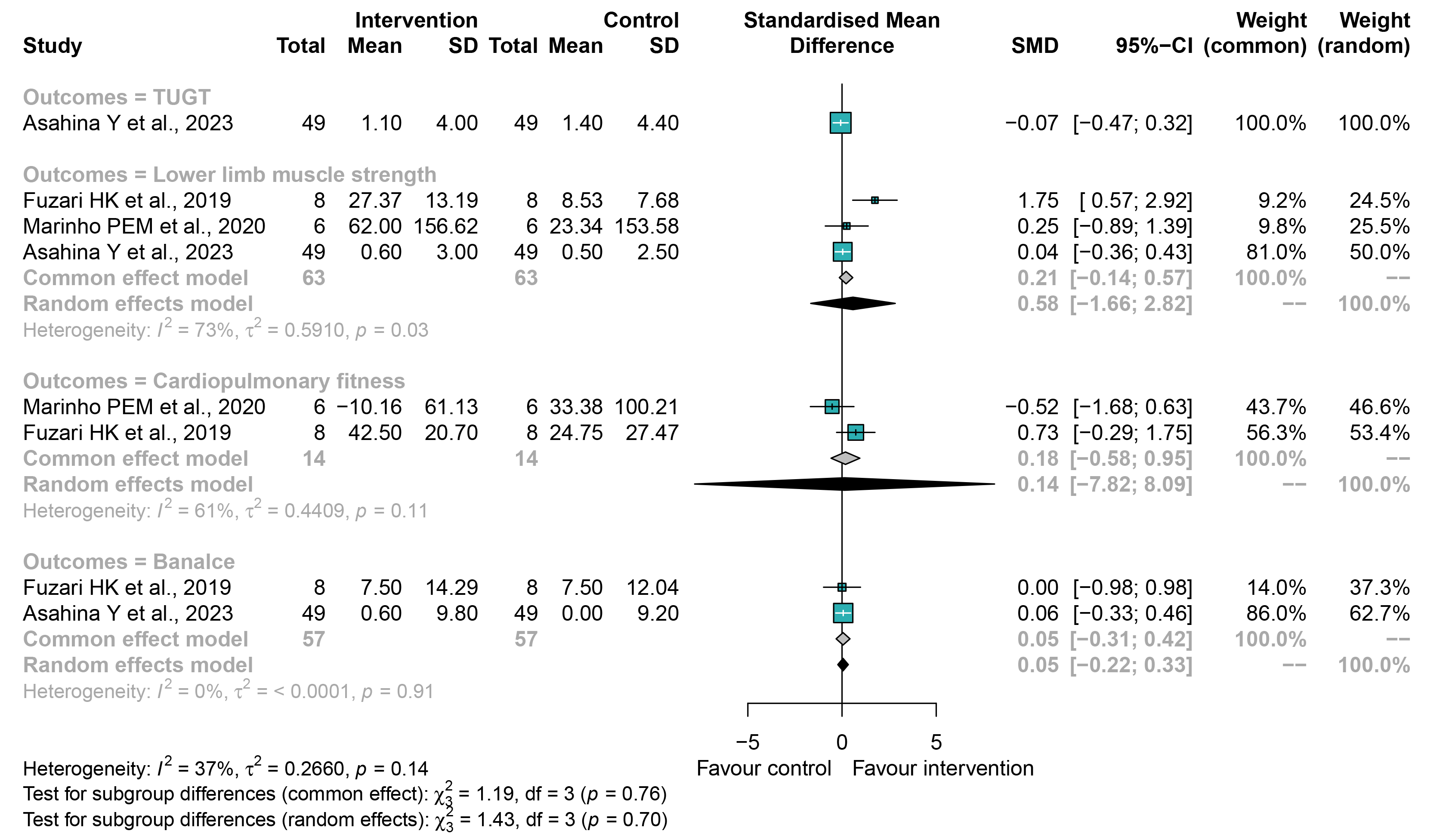


**Abbreviations:** SMD, standardized mean difference; CI, Confidence interval; TUGT, Time up and go test.

Reference

1. Seefried L, Genest F, Luksche N, Schneider M, Fazeli G, Brandl M, Bahner U, A AH: **Efficacy and safety of whole body vibration in maintenance hemodialysis patients - A pilot study**. *J Musculoskelet Neuronal Interact* 2017, **17**(4):268-274.

2. Doyle A, Chalmers K, Chinn DJ, McNeill F, Dall N, Grant CH: **The utility of whole body vibration exercise in haemodialysis patients: a pilot study**. *Clin Kidney J* 2017, **10**(6):822-829.

3. Fuzari HKB, de Andrade AD, Cerqueira MS, Pereira R, Medeiros AIC, Leite JC, Moura E, Souza HCM, Lima C, de Melo Marinho P: **Whole body vibration to attenuate reduction of explosive force in chronic kidney disease patients: a randomized controlled trial**. *Journal of exercise rehabilitation* 2018, **14**(5):883-890.

4. Fuzari HK, Dornelas de Andrade A, M AR, A IM, M FP, Lima AM, Cerqueira MS, Marinho PE: **Whole body vibration improves maximum voluntary isometric contraction of knee extensors in patients with chronic kidney disease: A randomized controlled trial**. *Physiother Theory Pract* 2019, **35**(5):409-418.

5. Maia TO, Paiva DN, Sobral Filho DC, Cavalcanti FCB, Rocha LG, Andrade CCA, Macedo Junior ARA, Marinho PEM: **Does whole body vibration training improve heart rate variability in kidney transplants patients? A randomized clinical trial**. *J Bodyw Mov Ther* 2020, **24**(2):50-56.

6. de Melo Marinho PE, da Rocha LG, de Araujo Filho JC, Sanudo B, Seixas A, de Andrade AD: **Is whole body vibration an alternative physical training method for renal transplant recipients?** *Physiother Res Int* 2020, **25**(3):e1838.

7. Yang YF, Huang CC, Chang CM, Lin HC: **Effect of Whole-Body Vibration Training on Physical Fitness and Postural Control in Working-Age Patients on Haemodialysis**. *J Rehabil Med Clin Commun* 2021, **4**:1000045.

8. Marinho PEM, Rocha LG, Araujo Filho JC, Araujo AXP, Andrade MDA, Taiar R, Paiva DN, Dornelas de Andrade A: **Effects of whole-body vibration on muscle strength, quadriceps muscle thickness and functional capacity in kidney transplant recipients: A randomized controlled trial**. *J Bodyw Mov Ther* 2021, **26**:101-107.

9. Asahina Y, Sakaguchi Y, Kajimoto S, Hattori K, Oka T, Kaimori JY, Kashihara N, Isaka Y: **A Randomized Controlled Trial of Whole-Body Vibration on Gait Ability and Balance among Older Hemodialysis Patients**. *Clin J Am Soc Nephrol* 2023, **18**(1):84-90.
